# Machine learning-based solar radiation prediction across heterogeneous climatic zones using NASA POWER data: a case study in Nigeria

**DOI:** 10.3389/frai.2026.1846084

**Published:** 2026-08-26

**Authors:** Oluwaseun Temitope Faloye, Grace Tina Awotoye, John Akinremi, Oluwatobi Solomon Olaleye, Ayoola Olamitomi Oluwadare, Oluwafemi E. Adeyeri, Laemthong Laokhongthavorn, Viroon Kamchoom

**Affiliations:** 1 Department of Water Resources Management and Agrometeorology, Federal University, Oye-Ekiti, Ekiti, Nigeria; 2 Federal College of Education Okene, Okene, Kogi, Nigeria; 3 Cranfied University Faculty of Engineering and Applied Science, Bedford, United Kingdom; 4 Department of Physical and Chemical Sciences, Faculty of Basic and Applied Sciences, Elizade University, Ilara-Mokin, Ondo, Nigeria; 5 ARC Centre of Excellence for the Weather of the 21st Century, Fenner School of Environment and Society, The Australian National University, Canberra, ACT, Australia; 6 Excellent Centre for Green and Sustainable Infrastructure, School of Engineering, King Mongkut’s Institute of Technology Ladkrabang, Bangkok, Thailand

**Keywords:** artificial intelligence, meteorological parameters, NASA power, Nigeria, solar radiation

## Abstract

Accurate prediction of solar radiation is essential for renewable energy planning, climate analysis, and agricultural productivity. Solar radiation data are required for the evaluation of solar power generation potential and crop water requirement computation, which is almost unavailable due to insufficient instrument for its measurement in many locations of the developing nations. Studies that applied several kernels of the Gaussian Process Regression (GPR) for the prediction of solar radiation are scarce. This study evaluates the performance of several machine learning models for solar radiation prediction across three climatic regions in Nigeria: Kano, Ibadan, and Onne. These locations represent distinct ecological zones ranging from the semi-arid savanna in northern Nigeria to the humid coastal rainforest in the southern region. Meteorological data used in this study were obtained from the NASA (National Aeronautics and Space Administration) POWER. Two machine learning approaches—Gaussian Process Regression (GPR) and Support Vector Machine (SVM)—were applied using different kernel configurations to predict solar radiation. Model performance was evaluated using standard statistical error metrics including Root Mean Square Error (RMSE), Mean Squared Error (MSE), Mean Absolute Error (MAE), Normalized Root Mean Square Error (NRMSE), and the coefficient of determination (*R*^2^). The results show that Gaussian Process Regression models generally produced the most accurate predictions across the study locations. In particular, the Exponential GPR model demonstrated the best performance in the humid regions of Ibadan and Onne, with RMSE values of 1.46 and 1.36 MJ.m^2^.d^−1^, respectively. Conversely, the Fine SVM model achieved the best prediction accuracy in Kano, recording the lowest RMSE value of 2.16 MJ.m^2^. d^−1^. These differences are attributed to variations in regional climatic conditions, where the relatively stable atmospheric conditions in the dry northern region favor SVM models, while the complex and highly variable atmospheric processes in humid regions are better captured by probabilistic models such as GPR. Overall, the findings highlight the importance of selecting appropriate machine learning models based on climatic characteristics when predicting solar radiation. The results provide useful insights for improving solar energy resource assessment and environmental modeling in Nigeria and similar tropical regions.

## 1 Introduction

Solar radiation is one of the most important environmental variables influencing both renewable energy production and agricultural productivity (Islam et al., 2009). As global energy demand continues to increase alongside concerns about climate change and environmental sustainability, solar energy has emerged as one of the most reliable and environmentally friendly renewable energy resources (Singh et al., 2011). Accurate estimation and prediction of solar radiation are therefore essential for a wide range of applications including solar power generation, agricultural water management, and climate modeling (Mellit and Kalogirou, 2008). In many regions of the world, especially in developing countries, the lack of dense ground-based radiation monitoring networks limits the availability of reliable solar radiation measurements. Consequently, predictive modeling techniques have become increasingly important for estimating solar radiation using available meteorological variables.

In agriculture, solar radiation plays a fundamental role in controlling several biophysical processes that determine crop growth and productivity. One of the most important agricultural applications of solar radiation is in the estimation of evapotranspiration, which represents the combined process of water evaporation from the soil surface and transpiration from plants. Solar radiation is a major input parameter in evapotranspiration models such as the FAO Penman–Monteith equation, where it directly influences the amount of energy available for water vaporization (Allen et al., 1998). Accurate estimation of evapotranspiration is critical for irrigation scheduling, water resource management, and improving agricultural productivity in water-limited environments. Solar radiation is also the primary energy source for photosynthesis, the process through which plants convert light energy into chemical energy to produce biomass. The portion of solar radiation that is usable by plants is referred to as Photosynthetically Active Radiation (PAR), which directly affects plant growth, crop yield, and ecosystem productivity (Jones, 2014). Variations in solar radiation due to atmospheric conditions such as cloud cover, humidity, and aerosols can therefore significantly influence agricultural performance. Reliable prediction of solar radiation can assist farmers and agricultural planners in understanding crop productivity patterns and optimizing agricultural management practices (Beer et al., 2010; Besharat et al., 2013; Ohunakin et al., 2015).

Beyond agriculture, solar radiation is the fundamental resource for solar photovoltaic (PV) power generation (Beer et al., 2010; Besharat et al., 2013; Ohunakin et al., 2015). Accurate solar radiation forecasting is essential for the design, installation, and efficient operation of solar energy systems. Reliable solar radiation data allow engineers and planners to estimate the potential energy output of photovoltaic systems and optimize energy generation strategies (Voyant et al., 2017). As many countries continue to expand their renewable energy portfolios, accurate solar radiation modeling has become increasingly important for sustainable energy planning.

Despite its importance, direct measurement of solar radiation requires specialized instruments such as pyranometers, which are often expensive to install and maintain in the developing nations, including Nigeria. As a result, many regions lack sufficient observational data. Satellite-based meteorological datasets provide an alternative source of reliable solar radiation data for locations where ground observations are limited. One widely used dataset is the NASA POWER, developed by NASA, which provides long-term global meteorological data suitable for renewable energy and agricultural applications.

In recent years, machine learning techniques have gained considerable attention for solar radiation prediction because of their ability to model complex nonlinear relationships among environmental variables (Li et al., 2012; Halabi et al., 2018; Makade et al., 2019; Huang et al., 2021). Traditional empirical and statistical models often struggle to capture the nonlinear interactions between meteorological parameters such as temperature, humidity, wind speed, and solar radiation (Besharat et al., 2013; Bhargawa and Singh, 2019). Machine learning algorithms, however, are capable of learning these complex patterns directly from data, thereby improving prediction accuracy (Voyant et al., 2017). Several artificial techniques approach like artificial neural network ANN, WT-LSTM, Support Vector Machine (SVM), Tree random forest algorithm and the gradient boosting regression tree (GBRT) algorithm have been used to predict the solar radiation and other similar parameters with encouraging results obtained (Sun et al., 2016; Persson et al., 2017; Fan et al., 2018; Zeng et al., 2020; Almawgani et al., 2025, 2026). In addition, several researchers have successfully applied deep learning technique for the prediction of solar radiation and other related parameters (Jayasankar et al., 2024; Vanlalchhuanawmi et al., 2025). The good result reported in the previous studies, and including the suitability of SVM and GPR with moderate data-set for solar radiation prediction informed the choice of SVM in this study, while two advantageous kernels were applied to advance knowledge. Moreover, previous studies did not consider the prediction of solar radiation in extremely contrasting climatic conditions to test the robustness of SVM model in predicting the solar radiation, which was consider for investigation in this study, thus revealing the innovation of the study. In addition, previous studies have scarcely considered the Gaussian Process Regression (GPR) for the prediction of solar radiation in extremely heterogeneous climatic zones, particularly in Nigeria. This study investigates the application of Gaussian Process Regression (GPR) alongside Support Vector Machine (SVM) for predicting global solar radiation across heterogeneous climatic zones in Nigeria using meteorological data obtained from NASA POWER, which is useful in areas with limited data availability like Nigeria.

Support Vector Machines, originally developed by Vladimir Vapnik, are powerful supervised learning algorithms that construct optimal regression functions based on statistical learning theory (Vapnik, 1998). Gaussian Process Regression, on the other hand, provides a probabilistic framework that models uncertainty and nonlinear relationships in environmental datasets (Rasmussen and Williams, 2006). These characteristics make both models suitable for solar radiation prediction under varying climatic conditions. Nigeria possesses significant solar energy potential due to its location within the tropical belt. However, solar radiation characteristics vary considerably across different ecological zones. Northern regions such as Kano experience semi-arid climatic conditions characterized by relatively low cloud cover and high solar radiation availability. In contrast, southern coastal regions such as Onne experience humid tropical climates with high cloud cover and atmospheric moisture, which can significantly influence solar radiation variability. Transitional regions such as Ibadan exhibit intermediate climatic characteristics. These climatic differences create spatial variations in solar radiation patterns that influence both agricultural productivity and solar energy generation potential. Identifying the model that could capture solar radiation spatial variability is critical for the accurate estimation of the solar radiation. Given the importance of solar radiation for evapotranspiration estimation, photosynthesis, and solar power generation, accurate modeling of solar radiation across different climatic regions is essential. Therefore, the objectives of this study are to (i) develop machine learning-based models for the prediction of solar radiation using NASA POWER meteorological data across the different climatic conditions in Nigeria; and (ii) evaluate the performance of Gaussian Process Regression and Support Vector Machine models for solar radiation prediction.

## 2 Materials and methods

### 2.1 Description of the study area

**Figure 1:**
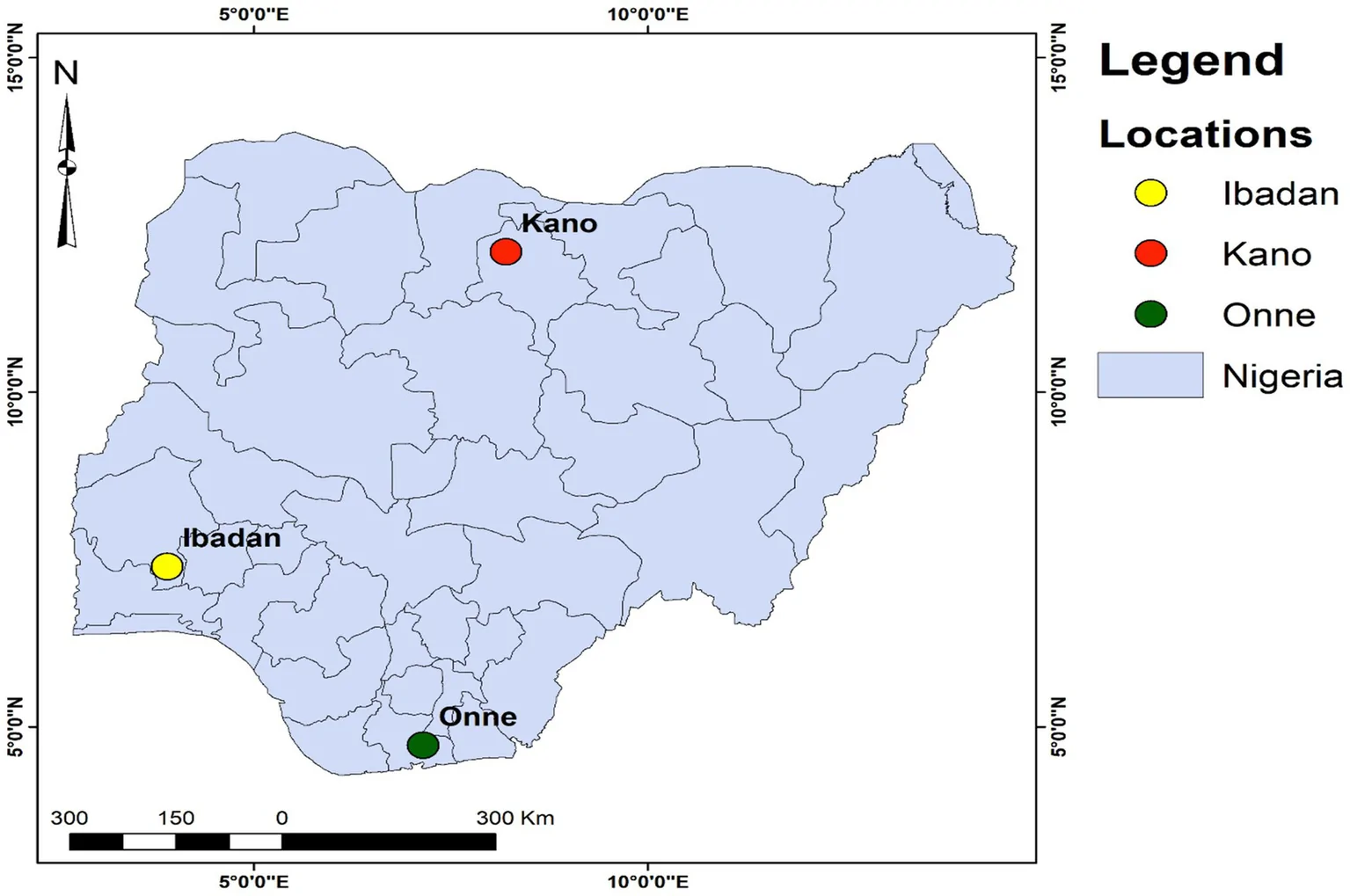
Map location of the study locations.

This study utilized meteorological data from three locations in Nigeria representing different climatic zones: Kano, Ibadan, and Onne. These locations were selected to represent the major ecological and climatic gradients in Nigeria, ranging from semi-arid conditions in the north to humid coastal environments in the south. The diversity in climatic characteristics across these locations makes them suitable for examining spatial variations in solar radiation and evaluating machine learning models for solar radiation prediction. Figure 1 showed the locations of the study area in Nigeria.

Kano is located in northern Nigeria within the Sudan savanna ecological zone. The region experiences a semi-arid tropical climate characterized by high temperatures, low relative humidity, and relatively low annual rainfall. The climate of Kano is marked by two distinct seasons: a dry season that typically lasts from November to April and a rainy season from May to October. The dry season is often influenced by the Harmattan wind, which originates from the Sahara Desert and brings dry and dusty air into the region. Due to its relatively clear skies and low cloud cover, Kano receives high levels of solar radiation throughout most of the year, making it a favorable location for solar energy applications (Akinsanola and Ogunjobi, 2014). These climatic characteristics also influence agricultural practices in the region, where irrigation is frequently required to sustain crop production.

Ibadan is located in southwestern Nigeria within the forest–savanna transitional ecological zone, approximately at latitude 7.4°N and longitude 3.9°E. The city experiences a tropical wet-and-dry climate characterized by moderate to high rainfall and relatively high humidity compared to northern Nigeria. Ibadan typically experiences a bimodal rainfall pattern, with two rainfall peaks occurring between April–July and September–October, separated by a short dry period commonly known as the “August break.” The presence of frequent cloud cover and higher atmospheric moisture content influences the amount of solar radiation reaching the Earth’s surface. As a transitional zone between the savanna and rainforest regions, Ibadan provides an intermediate climatic environment for studying solar radiation variability (Ayoade, 2003).

Onne is located in the Niger Delta region of southern Nigeria at approximately latitude 4.7°N and longitude 7.1°E. The area falls within the tropical rainforest climatic zone and is characterized by very high annual rainfall, high relative humidity, and persistent cloud cover throughout most of the year. Annual rainfall in the region often exceeds 2,000 mm, and the wet season typically extends from March to November, leaving only a short dry season between December and February. The proximity of Onne to the Atlantic Ocean contributes to strong maritime influences, resulting in high atmospheric moisture and frequent cloud formation. These climatic conditions significantly influence solar radiation variability and reduce the amount of solar radiation reaching the Earth’s surface compared to drier regions (NIMET, 2020).

The selection of these three locations therefore provides a representative coverage of Nigeria’s major climatic zones. This spatial diversity allows for a comprehensive assessment of solar radiation variability across different environmental conditions and enables the evaluation of machine learning models under varying atmospheric dynamics. Understanding these regional differences is important for improving solar radiation prediction for applications in renewable energy planning, agricultural water management, evapotranspiration estimation, and crop productivity modeling.

### 2.2 Meteorological data collection for the three locations

Meteorological data (average monthly per day) used in this study were obtained from the NASA POWER developed by NASA. The NASA POWER database provides long-term global meteorological and solar energy parameters derived from satellite observations and atmospheric reanalysis products, making it a reliable data source for environmental modeling, agricultural studies, and renewable energy assessments (Stackhouse et al., 2018). The dataset is widely used in scientific research because it provides consistent and continuous meteorological information for locations where ground-based measurements may be limited. For this study, several key meteorological variables that influence solar radiation variability were extracted from the NASA POWER dataset. These variables include cloud amount, all-sky surface total Photosynthetically Active Radiation (PAR), maximum air temperature, minimum air temperature, and relative humidity. Cloud amount is an important atmospheric parameter that directly affects the amount of solar radiation reaching the Earth’s surface by influencing atmospheric attenuation and scattering processes. Increased cloud cover generally reduces solar radiation availability, while clear-sky conditions allow greater solar energy to reach the surface.

The all-sky surface total Photosynthetically Active Radiation (PAR) parameter represents the portion of solar radiation within the wavelength range of approximately 400–700 nm that is available for plant photosynthesis. PAR is an important variable in agricultural and ecological studies because it directly influences plant growth, biomass production, and crop yield (Jones, 2014). In addition, PAR plays a significant role in estimating ecosystem productivity and evaluating agricultural performance under varying climatic conditions. Air temperature variables, including maximum and minimum air temperature, are also critical meteorological parameters influencing atmospheric processes and solar radiation dynamics. Temperature affects atmospheric stability, cloud formation, and evaporation processes, which can indirectly influence solar radiation availability. Similarly, relative humidity is an important atmospheric parameter that reflects the amount of moisture present in the air. High relative humidity is often associated with increased cloud formation and atmospheric water vapor, both of which can significantly affect solar radiation transmission through the atmosphere (Ayoade, 2003). While the meteorological variables used as input parameters in this study were obtained from the NASA POWER^1^ dataset between 1984 and 2007 (24 years), the observed solar radiation data for the study locations were obtained from ground-based weather stations operated by the Nigerian Meteorological Agency (NiMET) between 1984 and 2007 (24 years). These stations are located in Kano, Ibadan, and Onne. The NiMET weather stations provide reliable in-situ measurements of solar radiation using calibrated meteorological instruments such as pyranometers. The combination of satellite-derived meteorological variables from NASA POWER and ground-measured solar radiation data from NiMET provides a robust dataset for evaluating machine learning models for solar radiation prediction. Using satellite data ensures the availability of consistent meteorological information across different locations, while ground-based observations provide accurate reference values for model training and validation.

### 2.3 Development of the machine learning algorithms

Machine learning algorithms were developed to predict solar radiation using selected meteorological parameters obtained from the NASA POWER provided by NASA. The input variables used for model development included cloud amount, all-sky surface total photosynthetically active radiation (PAR), maximum air temperature, minimum air temperature, and relative humidity. These variables are known to influence solar radiation variability and are commonly used in solar radiation modeling studies (Voyant et al., 2017). The machine learning models were designed to learn the nonlinear relationships between these meteorological parameters and the observed solar radiation values obtained from the weather stations of the Nigerian Meteorological Agency. A total of 864 datasets were used for the modeling process. To ensure effective model development and reliable evaluation of prediction performance, the dataset was divided into two subsets consisting of 70% for model training and 30% for model validation. To ensure the model capture the variations in the climatic variations well in the prediction, 70% of the data were extracted from each location and pool together during training, while 30% of the same was repeated during training. In each location, data used for training ranged between 1984 and 2000, while the data used for model validation ranged between year 2000 and 2007. To minimize sampling bias, the data combination procedure during training and validation ensured that both subsets contained representative samples from all three climatic zones. Furthermore, duplicate observations were not permitted between the training and testing datasets, thereby preventing data leakage. The training dataset was used to build and calibrate the machine learning models, while the validation dataset was used to assess the predictive accuracy and generalization capability of the models. This training–validation approach is widely used in machine learning studies to prevent model overfitting and to ensure that the developed models perform well on unseen data (Goodfellow et al., 2016).

Two major machine learning approaches were employed in this study: Support Vector Machine (SVM) and Gaussian Process Regression (GPR). Support Vector Machine is a supervised learning algorithm based on statistical learning theory developed by Vladimir Vapnik. SVM models are widely used for regression and classification tasks due to their ability to handle nonlinear relationships and produce high prediction accuracy with relatively small datasets (Vapnik, 1998). In this study, two SVM variants were implemented: Fine SVM and Medium SVM. The Fine SVM model uses a smaller kernel scale that allows the algorithm to capture finer details and subtle patterns within the dataset, while the Medium SVM model uses a moderately sized kernel scale that balances model flexibility and generalization. The MATLAB tool box works such that it optimizes the prediction, and produced kernel scale of 0.56 and 2.2 for fine and medium gaussian SVM, while the box constraint and epsilon were automatic.

In addition to SVM models, several Gaussian Process Regression (GPR) models were developed to evaluate their predictive performance for solar radiation estimation. GPR is a non-parametric probabilistic machine learning approach that models complex nonlinear relationships by defining a Gaussian distribution over possible functions that fit the data. Support Vector Machine and Gaussian Process Regression were selected because they exhibit strong predictive performance on relatively small and moderately sized datasets (864 data points) while maintaining good generalization capability and computational efficiency (Hossain et al., 2022). Moreover, several studies have applied similar or lower number of data point for successful ML prediction in environmental studies (Nikam et al., 2010; Faloye et al., 2025a, 2025b). Sophisticated methods such as Random Forest, Extreme Gradient Boosting, Long Short-Term Memory (LSTM), and Transformer-based deep learning models have recently demonstrated excellent forecasting performance, but these approaches generally require considerably larger datasets, higher computational resources, and more extensive hyperparameter optimization (Hossain et al., 2022).

In addition, one of the advantages of GPR is its ability to provide both predictive mean values and uncertainty estimates for predictions, making it particularly useful for environmental modeling applications (Rasmussen and Williams, 2006). The GPR models implemented in this study included Exponential GPR, Squared Exponential GPR, Rational Quadratic GPR, and Matern 5/2 GPR kernels. Each kernel function characterizes different smoothness properties of the regression function, allowing the models to capture different levels of variability in the dataset. The kernel scale, kernel sigma and sigma were automatic. Hyperparameter optimization was performed using MATLAB Regression Learner’s built-in Bayesian optimization framework. For SVM, kernel scale and box constraint parameters were optimized by minimizing cross-validation loss. For GPR, kernel function parameters and noise variance were similarly optimized using Bayesian optimization with five-fold cross-validation under MATLAB’s default optimization settings.

The development, training, and validation of all machine learning models were carried out using MATLAB version 2019a, which provides a comprehensive machine learning toolbox for regression analysis and predictive modeling. MATLAB offers built-in functions for training SVM and GPR models, optimizing hyperparameters, and evaluating model performance using statistical error metrics. By comparing the performance of different SVM and GPR models, this study aims to identify the most suitable machine learning approach for solar radiation prediction across different climatic zones in Nigeria. The models were subsequently evaluated using standard statistical performance indicators to determine their accuracy, reliability, and predictive capability.

### 2.4 Theory of support vector machine (SVM) and Gaussian process regression (GPR)

#### 2.4.1 Support vector machine (SVM)

Support Vector Machine (SVM) is a supervised machine learning algorithm based on the principles of statistical learning theory developed by Vladimir Vapnik. Although SVM was originally designed for classification problems, it has been widely extended to regression problems, commonly referred to as Support Vector Regression (SVR). The fundamental idea behind SVM is to determine an optimal hyperplane that best represents the relationship between input variables and the target output while minimizing prediction errors (Vapnik, 1998). In regression applications, SVM attempts to find a function that approximates the relationship between the input variables and the target variable with a specified tolerance level. One of the key features of SVM is the use of kernel functions, which enable the algorithm to model nonlinear relationships without explicitly transforming the data into higher-dimensional space. Common kernel functions include linear, polynomial, radial basis function (RBF), and Gaussian kernels. The kernel function allows SVM to effectively capture complex nonlinear relationships between meteorological parameters and solar radiation.

Another important concept in SVM is the *ε*-insensitive loss function, which defines a margin of tolerance within which prediction errors are not penalized. Only data points that fall outside this margin contribute to the optimization process, and these data points are known as support vectors. These support vectors are critical elements that define the regression function and determine the position of the hyperplane. SVM is particularly effective for datasets with complex nonlinear patterns and relatively small sample sizes because it focuses only on a subset of critical training points (support vectors), thereby improving computational efficiency and generalization capability (Smola and Schölkopf, 2004).

#### 2.4.2 Gaussian process regression (GPR)

Gaussian Process Regression (GPR) is a probabilistic, non-parametric machine learning technique used for regression and prediction tasks. Unlike traditional regression methods that assume a specific functional form, GPR defines a probability distribution over possible functions that fit the observed data. This allows the model to capture complex nonlinear relationships while also providing a measure of uncertainty for predictions (Rasmussen and Williams, 2006). A Gaussian process can be defined as a collection of random variables, any finite number of which follow a joint Gaussian distribution. In GPR, the relationship between input variables and the output variable is modeled using a mean function and a covariance function (kernel).

The covariance function plays a crucial role in determining the behavior of the regression model. Different kernel functions allow the model to capture different patterns in the data. Common kernels used in GPR include the Squared Exponential kernel, Exponential kernel, Rational Quadratic kernel, and Matern kernel. These kernels define how closely related two data points are based on their distance in the input space. One major advantage of GPR is that it provides both predictive mean values and predictive variance, which quantifies the uncertainty associated with each prediction. This characteristic makes GPR particularly useful for environmental and atmospheric modeling, where uncertainty in the data is often significant. Furthermore, GPR is highly flexible and capable of modeling complex nonlinear relationships between meteorological variables and solar radiation. Because the model is probabilistic, it can adapt to noisy data and varying atmospheric conditions, making it suitable for applications such as solar radiation prediction, climate modeling, and environmental forecasting.

### 2.5 Model statistical evaluation indices

To evaluate the predictive performance of the developed machine learning models for solar radiation estimation, several statistical evaluation indices were used. These indices provide quantitative measures of the difference between the observed solar radiation values obtained from the weather stations of the Nigerian Meteorological Agency and the predicted values generated by the machine learning models. The performance of the models was assessed using Root Mean Square Error (RMSE), Normalized Root Mean Square Error (NRMSE), Mean Squared Error (MSE), and Mean Absolute Error (MAE) (Equations 1–4). These statistical indicators are commonly used in environmental modeling and renewable energy studies to assess the accuracy and reliability of prediction models (Willmott and Matsuura, 2005; Chai and Draxler, 2014).

$$\text{RMSE}=\sqrt{1/n\sum {\left(M_{i}-S_{i}\right)}^{2}}$$ (1)

$$\text{NRMSE}=\frac{\sqrt{1/n\sum {\left(M_{i-}\;S_{i}\right)}^{2}}}{\bar{M}}$$ (2)

$$\mathrm{MSE}=\frac{1}{n}\sum_{i=n}^{n}|M_{i}-S_{i}|$$ (3)

$$\mathrm{MSE}=\frac{1}{n}\sum_{i=n}^{n}{|M_{i}-S_{i}|}^{2}$$ (4)

where *M_i_* and *S_i_* are measured and predicted data, respectively. $\bar{M}$ is the mean value of *M_i_*, and *n* is the number of observations.

As the Normalized Root Mean Square Error (NRMSE) approaches zero, the accuracy of the model improves. Based on established classification criteria, a model is considered excellent when the NRMSE is less than or equal to 10% (NRMSE ≤ 0.1), good when it lies between 10 and 20% (0.1 < NRMSE ≤ 0.2), and fair when it falls between 20 and 30% (0.2 < NRMSE ≤ 0.3). However, when the NRMSE exceeds 30% (NRMSE > 0.3), the model performance is regarded as poor (Jamieson et al., 1991). Furthermore, the predictive accuracy of a model increases as other statistical error indicators such as Mean Squared Error (MSE), Mean Absolute Error (MAE), and Root Mean Square Error (RMSE) approach zero. The precision of the model was also evaluated using the coefficient of determination (*R*^2^), where values closer to 1 indicate a stronger agreement between the predicted and observed values, signifying better model performance.

## 3 Results and discussions

### 3.1 Spatio-temporal trend of all-sky surface total PAR and cloud amount across the locations

The analysis of satellite-derived climatic variables from the NASA POWER provides important insights into the temporal trends of all-sky surface Photosynthetically Active Radiation (PAR) and cloud amount across three major Nigerian locations: Kano, Ibadan, and Onne. These locations represent distinct climatic zones in Nigeria, ranging from the semi-arid Sudan savanna in northern Nigeria (Kano), through the transitional rainforest–savanna zone (Ibadan), to the humid coastal rainforest environment in the Niger Delta (Onne). Such climatic differences strongly influence solar radiation availability and atmospheric conditions across these regions.

**Figure 2:**
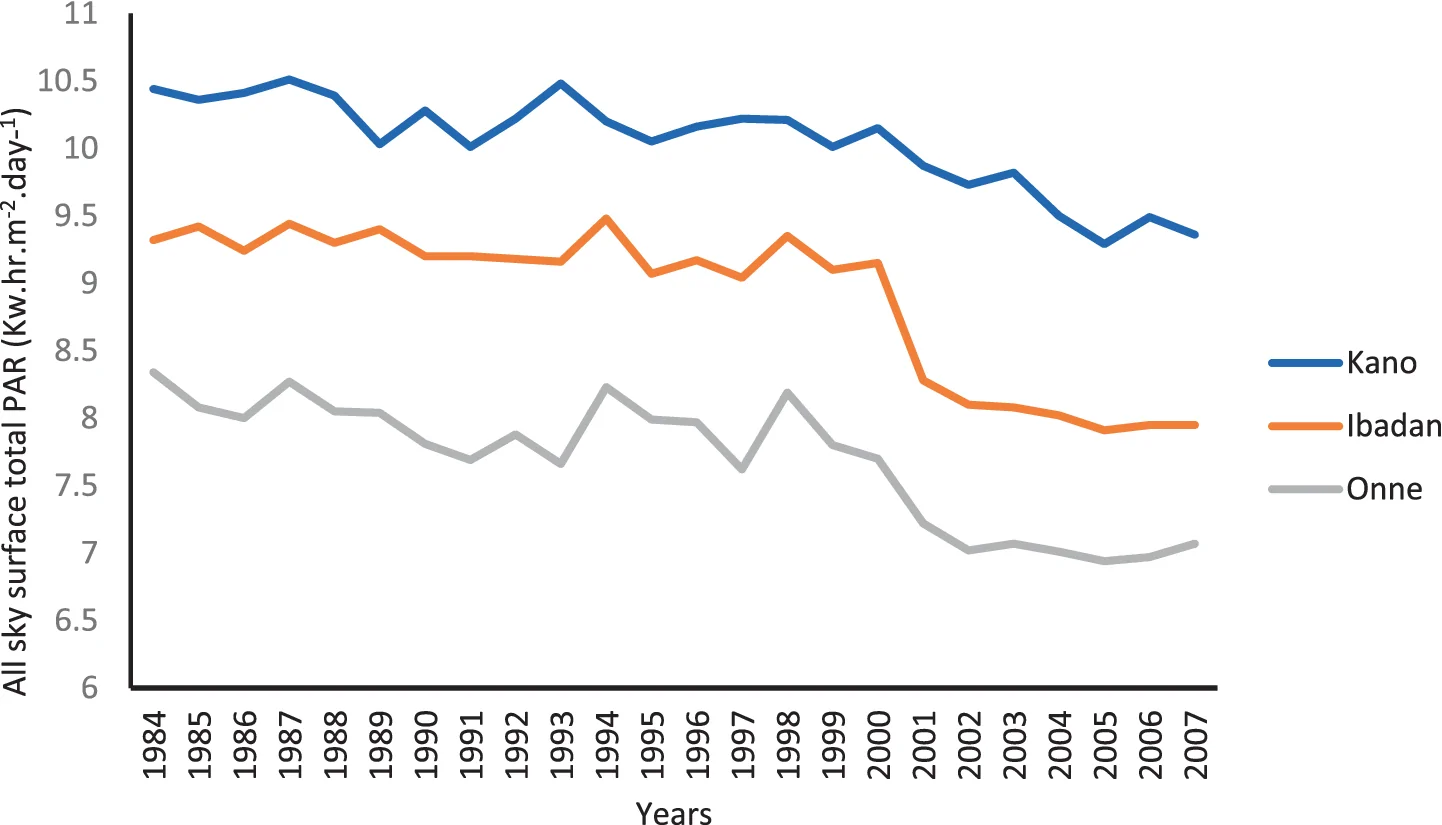
Relationship between the NASA POWER all-sky surface total PAR and years for all locations.

Figure 2 illustrates the temporal relationship between all-sky surface PAR and years for the three locations. PAR refers to the portion of incoming solar radiation within the 400–700 nm wavelength band that is usable for plant photosynthesis, commonly known as Photosynthetically Active Radiation. As a key driver of plant growth and ecosystem productivity, variations in PAR directly affect vegetation development, agricultural productivity, and carbon cycling (Monteith and Unsworth, 2013). Figure 2 indicates noticeable interannual fluctuations in PAR across the study period, suggesting that solar radiation availability varies over time due to changes in atmospheric conditions such as cloud cover, humidity, and aerosol concentrations. Among the three locations, Kano generally exhibits relatively higher PAR values compared with Ibadan and Onne. This pattern can be attributed to Kano’s location in the semi-arid northern region of Nigeria, where cloud cover is typically lower and atmospheric moisture is limited. Reduced cloudiness allows greater penetration of solar radiation to the Earth’s surface, resulting in increased PAR availability. Previous studies have shown that semi-arid regions often experience higher levels of solar radiation due to clearer atmospheric conditions and lower humidity (Jones, 2014). Consequently, vegetation in northern Nigeria often receives higher radiation input during the growing season compared with more humid southern regions.

In contrast, Onne tends to exhibit comparatively lower PAR values, which can be explained by its coastal location within the humid Niger Delta region. This area is characterized by persistent cloud cover, high humidity, and frequent rainfall associated with the West African monsoon system. These atmospheric conditions reduce the transmission of solar radiation reaching the surface because clouds reflect and scatter incoming radiation back into the atmosphere (Wild, 2012). As a result, less radiation within the PAR spectrum reaches plant canopies in this region. Ibadan displays intermediate PAR levels, reflecting its transitional climatic position between the dry northern savanna and the humid southern rainforest zones. The climate of Ibadan is influenced by both maritime and continental air masses, leading to moderate cloud cover and seasonal variability in solar radiation. During the dry season, when cloud cover is reduced, PAR levels may increase, while during the rainy season the presence of clouds tends to decrease radiation availability.

**Figure 3:**
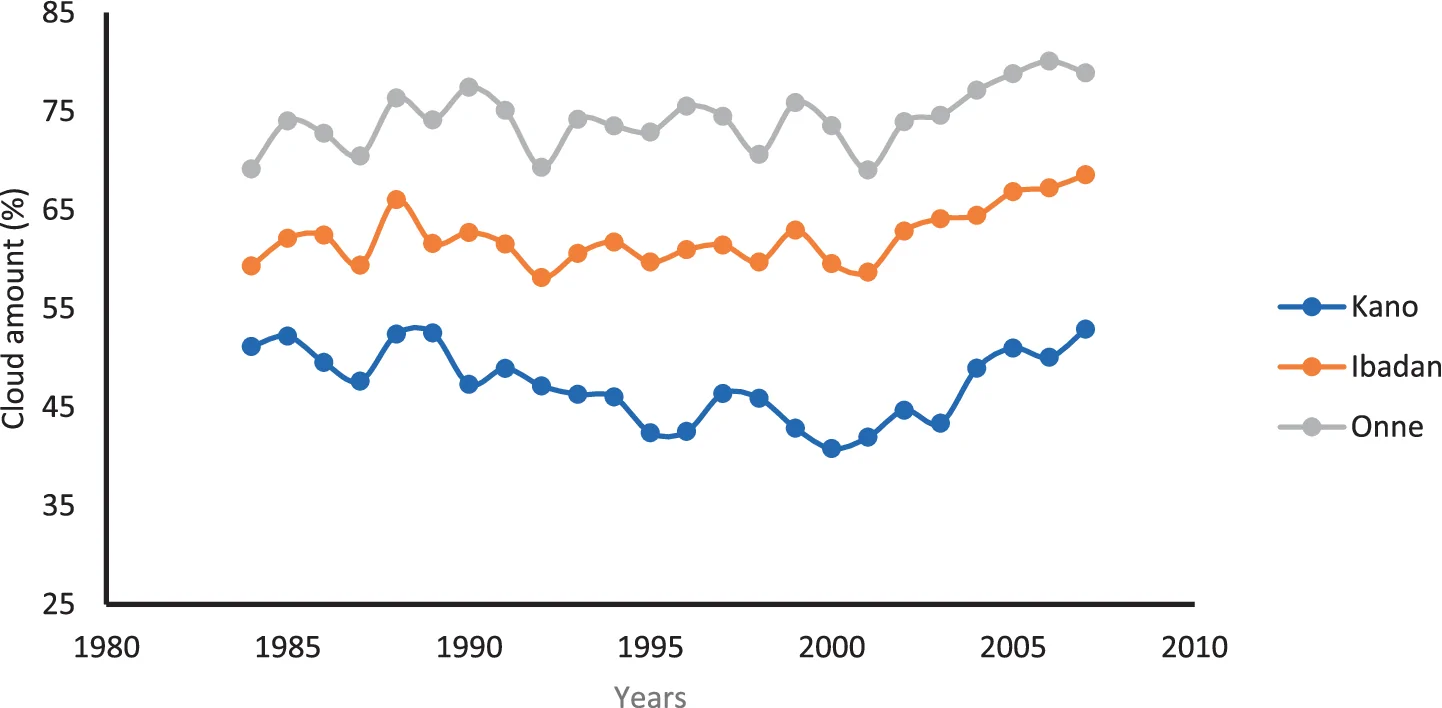
Relationship between NASA POWER cloud amounts and years for all locations.

Figure 3 further highlights the temporal variation in cloud amount across the three locations. The results reveal fluctuations in cloud cover over time, which strongly influence the availability of solar radiation and PAR. Among the three study areas, Onne consistently records higher cloud amounts, reflecting its humid tropical climate and proximity to the Atlantic Ocean. High evaporation rates and moisture transport from the ocean contribute to frequent cloud formation in the region (Adefolalu, 2007). These persistent clouds reduce the amount of solar radiation reaching the Earth’s surface, thereby lowering PAR levels.

In contrast, Kano typically exhibits lower cloud amounts, consistent with its semi-arid climatic conditions. The reduced atmospheric moisture and lower frequency of precipitation events in northern Nigeria limit cloud formation. Consequently, clearer skies allow greater solar radiation penetration, resulting in higher PAR availability. This relationship between low cloud cover and increased solar radiation has been widely documented in radiation balance studies (Monteith and Unsworth, 2013). Ibadan again shows moderate cloud cover, reflecting its location within the forest–savanna transition zone. Seasonal atmospheric circulation patterns, particularly the movement of the Inter-Tropical Discontinuity (ITD), influence cloud formation and rainfall distribution in this region. During the wet season, moist southwesterly monsoon winds increase cloud cover, whereas the dry season is characterized by the dominance of dry northeasterly Harmattan winds that reduce cloudiness. The inverse relationship between cloud amount and PAR observed across the three locations is consistent with established atmospheric radiation theory. Clouds play a critical role in regulating the Earth’s radiation balance by reflecting incoming solar radiation and absorbing part of the energy before it reaches the surface. As cloud cover increases, the amount of solar radiation available within the PAR spectrum decreases. Conversely, reduced cloudiness enhances the transmission of solar radiation and increases PAR availability (Wild, 2012).

Overall, the observed trends demonstrate clear spatial differences in solar radiation distribution across Nigeria’s climatic zones. Northern regions such as Kano benefit from relatively higher radiation availability due to lower cloud cover, while southern coastal regions like Onne experience reduced radiation due to persistent atmospheric moisture and cloud formation. Ibadan occupies an intermediate position with moderate radiation levels and seasonal variability. Understanding these patterns is essential for climate research, agricultural planning, and ecosystem productivity assessment. Since PAR directly influences photosynthesis and plant growth, variations in radiation availability across these regions may affect crop yields, vegetation dynamics, and carbon exchange processes. Satellite-derived datasets such as the NASA POWER system therefore provide valuable tools for monitoring climatic variability and supporting environmental research in regions where ground-based meteorological observations are limited.

### 3.2 Spatio-temporal trend of relative humidity across the locations

**Figure 4:**
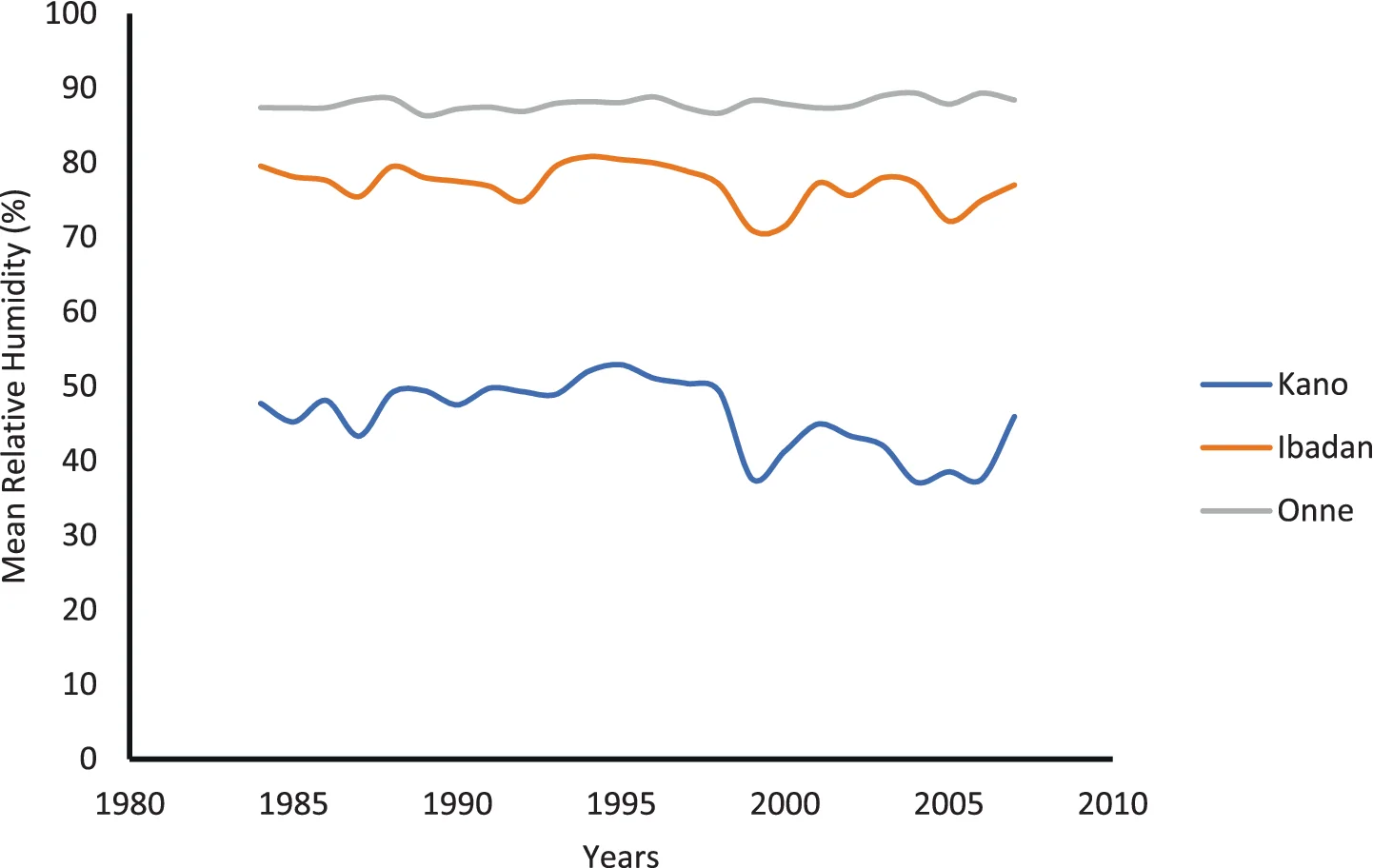
Relationship between NASA POWER mean relative humidity and years for all locations.

Figure 4 further highlights the trend in mean relative humidity across the three locations. Relative humidity plays a crucial role in atmospheric processes because it influences cloud formation, precipitation, and radiation balance. The results suggest that Onne experiences the highest levels of relative humidity due to its coastal location and strong maritime influence. High humidity levels promote cloud formation and reduce atmospheric transparency, which in turn limits solar radiation reaching the surface.

Ibadan exhibits moderate relative humidity levels that fluctuate seasonally in response to changes in atmospheric circulation patterns. Meanwhile, Kano records the lowest relative humidity values, reflecting its semi-arid climatic conditions. Lower humidity levels are associated with clearer skies and increased solar radiation penetration, further explaining the higher PAR values observed in the region. Overall, the trends observed across Kano, Ibadan, and Onne demonstrate clear spatial and temporal differences in atmospheric conditions across Nigeria’s climatic zones. The northern savanna region represented by Kano tends to experience higher solar radiation and lower humidity due to reduced cloud cover, whereas the southern coastal region.

represented by Onne experiences lower radiation levels because of persistent atmospheric moisture and cloudiness. Ibadan occupies an intermediate position characterized by moderate radiation availability and seasonal variability. These findings highlight the importance of satellite-based climate datasets such as the NASA POWER system in understanding regional climatic patterns. Reliable long-term radiation and meteorological data are essential for agricultural planning, climate change research, and ecosystem productivity assessment. Since PAR is a primary driver of plant photosynthesis, variations in radiation availability across these regions may significantly influence crop productivity, vegetation growth, and carbon cycling processes.

### 3.3 Spatio-temporal trend of maximum and minimum air temperature, and solar radiation across the locations

**Figure 5:**
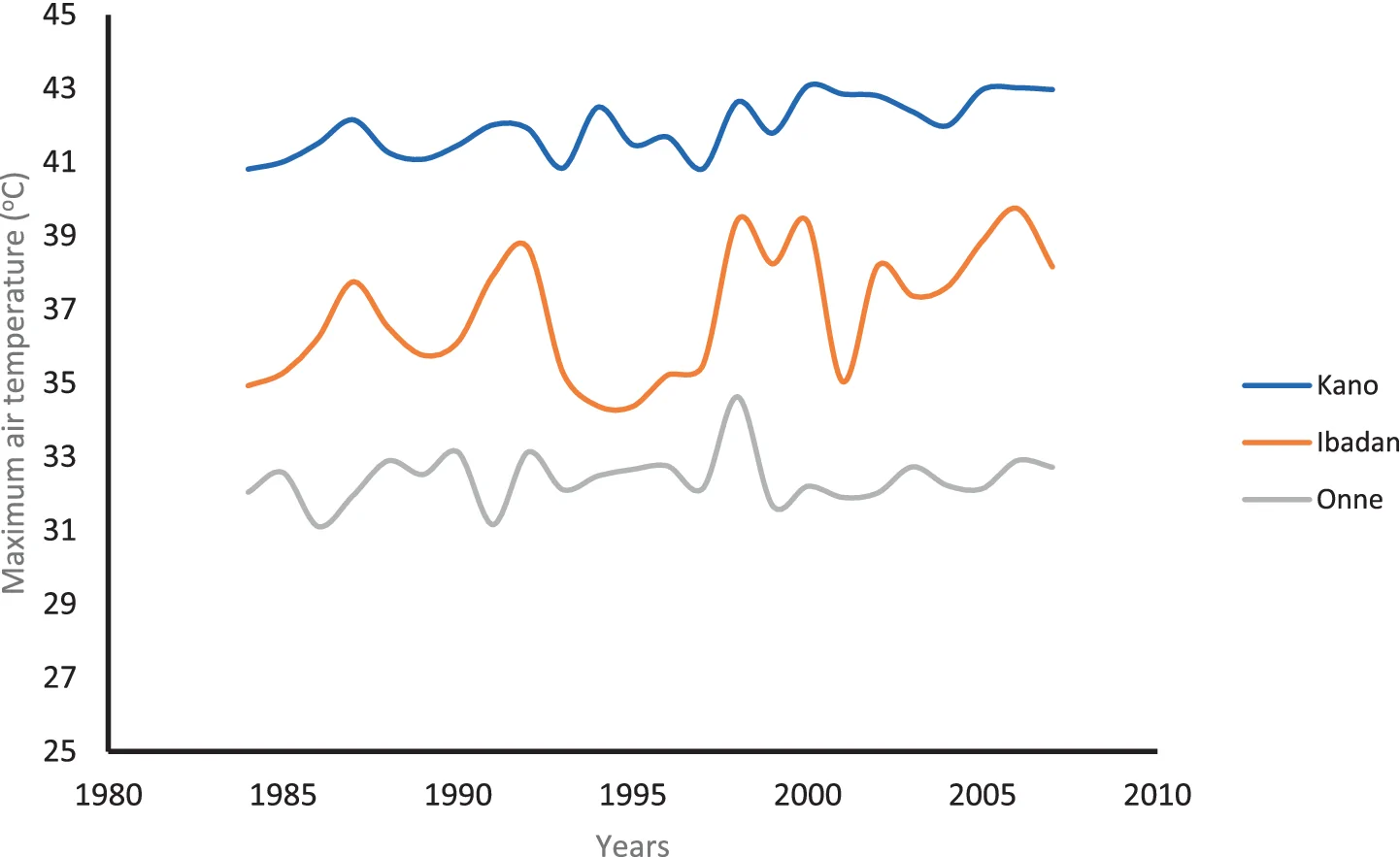
Relationship between NASA POWER maximum air temperature and years for all locations.

**Figure 6:**
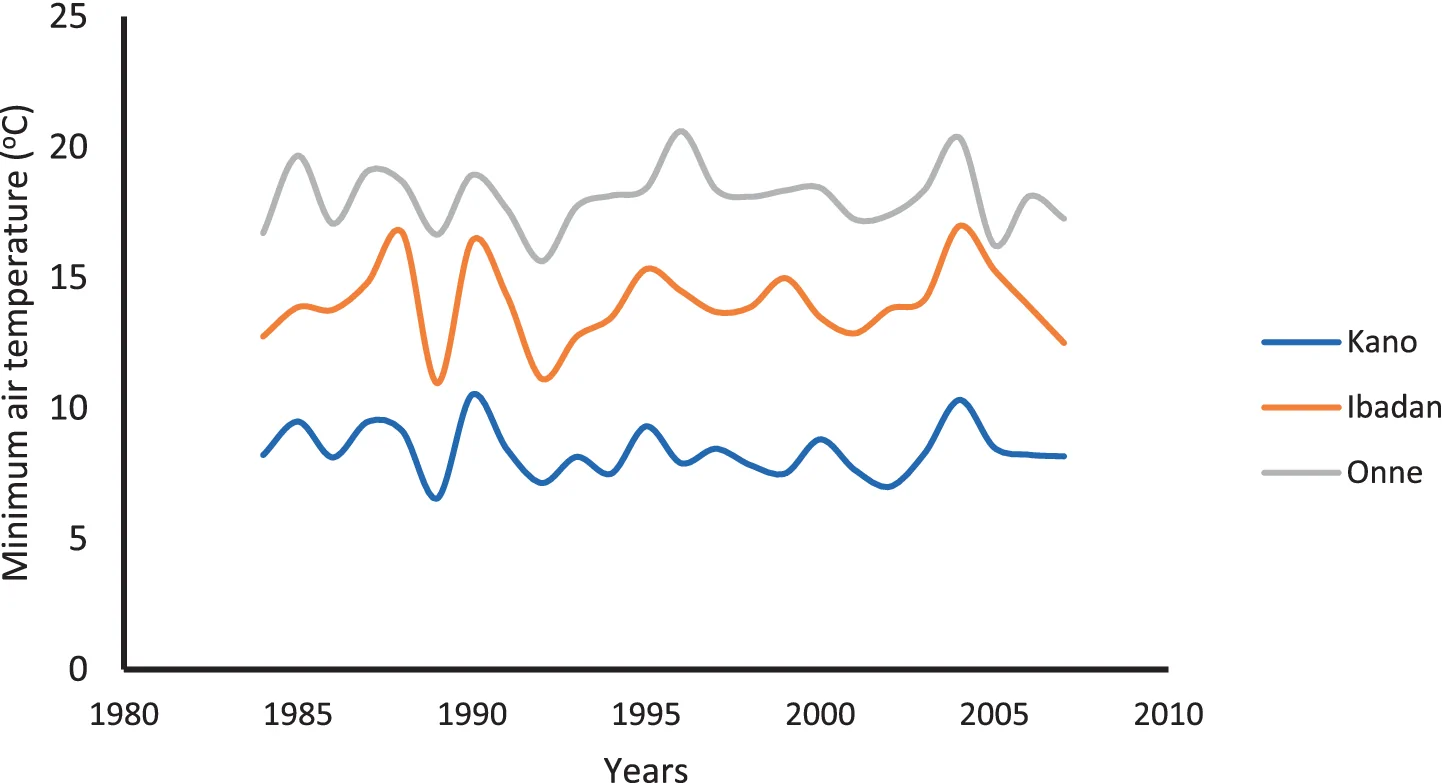
Relationship between NASA POWER minimum air temperature and years for all locations.

**Figure 7:**
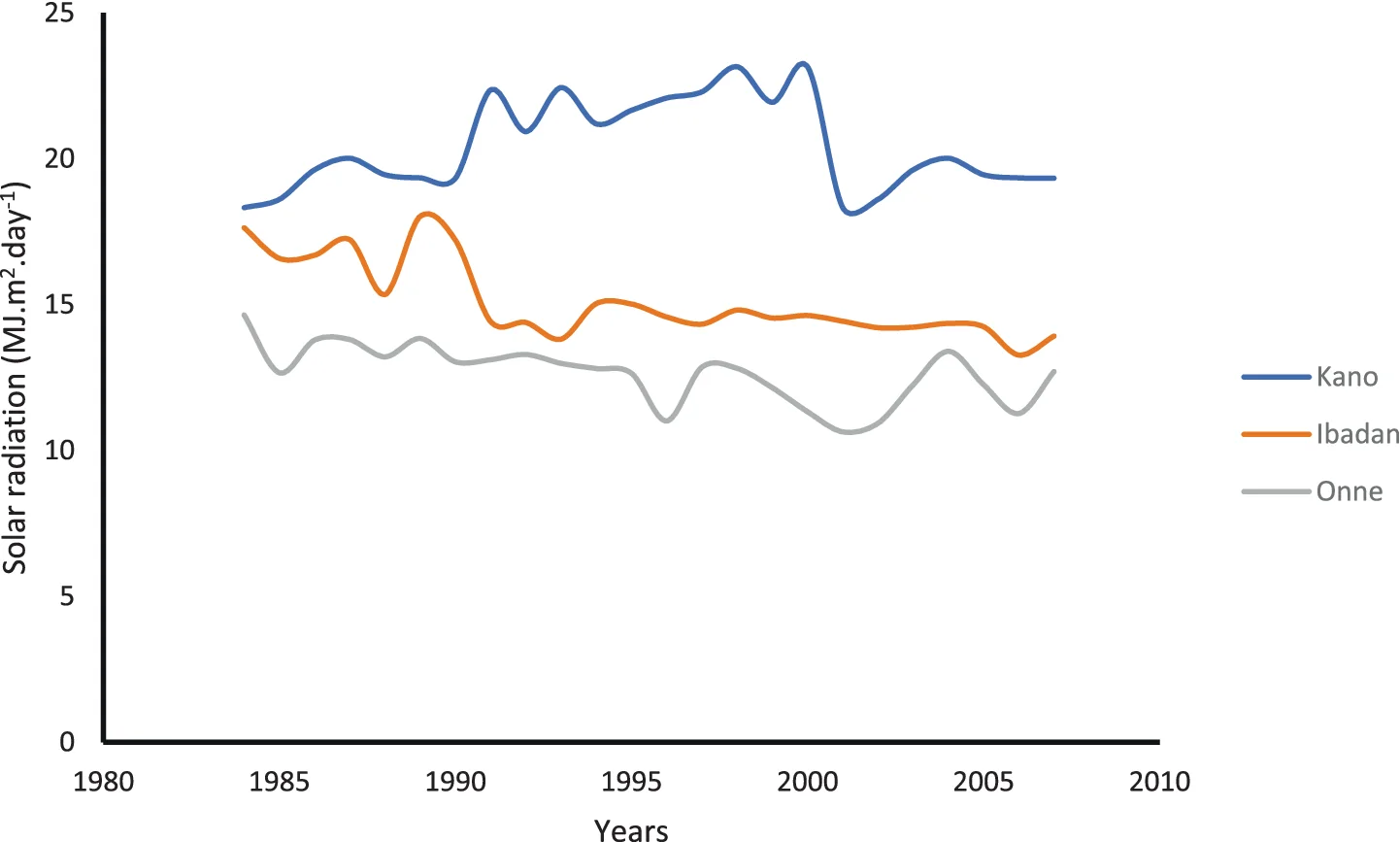
Relationship between measured solar radiation and years for all locations.

The climatic variables presented in Figures 5–7 illustrate the long-term trends of maximum air temperature, minimum air temperature, and measured solar radiation across three Nigerian locations: Kano, Ibadan, and Onne. These data were obtained from the NASA POWER, which provides long-term satellite-derived meteorological and radiation parameters for environmental and climate studies. The three locations represent distinct climatic zones in Nigeria, including the semi-arid Sudan savanna (Kano), the forest–savanna transition zone (Ibadan), and the humid coastal rainforest region (Onne). These ecological differences play an important role in shaping temperature and radiation variability across the country.

Figure 5 illustrates the relationship between maximum air temperature and years across the three study locations. The trend indicates noticeable interannual variability in maximum temperature over the study period. In general, Kano records relatively higher maximum temperatures compared with Ibadan and Onne. This observation is consistent with the climatic characteristics of northern Nigeria, where lower cloud cover and reduced atmospheric moisture allow greater solar radiation to reach the Earth’s surface, leading to higher daytime heating. Semi-arid environments typically experience higher maximum temperatures because clear skies enhance the absorption of incoming solar radiation by the land surface (Monteith and Unsworth, 2013).

In contrast, Onne tends to exhibit relatively lower maximum temperature values. The region is located in the humid Niger Delta and experiences high rainfall, dense cloud cover, and strong maritime influence from the Atlantic Ocean. These atmospheric conditions reduce the intensity of incoming solar radiation reaching the surface and moderate daytime heating. Cloud cover acts as a reflective barrier that limits solar radiation penetration, thereby lowering maximum temperature values in coastal regions (Wild, 2012). Ibadan shows intermediate maximum temperature levels between Kano and Onne. The city lies within the forest–savanna transition zone and experiences climatic conditions influenced by both continental and maritime air masses. Seasonal variations in the West African monsoon system affect temperature patterns in this region. During the dry season, reduced cloud cover allows temperatures to rise, while increased cloudiness during the rainy season moderates daytime heating.

Figure 6 presents the trend of minimum air temperature across the study locations. Minimum temperature represents minimum air temperature conditions and is strongly influenced by atmospheric moisture and cloud cover. Figure 6 suggests gradual fluctuations in minimum temperature over time across the three locations. In general, Onne tends to record relatively higher minimum temperatures compared with Kano. This pattern can be attributed to the high humidity and cloud cover in the coastal region. Clouds trap outgoing longwave radiation emitted by the Earth’s surface during the night, thereby preventing rapid cooling and maintaining higher nighttime temperatures (Jones, 2014).

Conversely, Kano often records lower minimum temperatures, especially during the dry season. The relatively clear skies and low atmospheric moisture allow greater radiative heat loss from the Earth’s surface at night. As a result, surface temperatures cool more rapidly compared with humid regions. This phenomenon is typical of semi-arid climates, where large diurnal temperature ranges occur due to strong daytime heating and rapid nighttime cooling. Ibadan again exhibits moderate minimum temperature values, reflecting its transitional climatic position. The combination of moderate humidity levels and seasonal cloud cover results in nighttime temperatures that are generally higher than those observed in Kano but lower than those recorded in Onne.

Figure 7 illustrates the trend in measured solar radiation across the three locations. Solar radiation plays a critical role in the Earth’s energy balance and is closely related to plant productivity through the process of Photosynthetically Active Radiation. The figure shows variability in solar radiation across the study period, reflecting changes in atmospheric conditions such as cloud cover, humidity, and aerosol concentration.

Among the three locations, Kano generally records higher solar radiation values, which corresponds with its lower cloud cover and drier atmospheric conditions. Clear skies allow more solar radiation to reach the Earth’s surface, resulting in greater radiation availability. This finding aligns with previous studies indicating that semi-arid regions often experience higher solar radiation levels due to reduced atmospheric scattering and absorption (Monteith and Unsworth, 2013). In contrast, Onne records comparatively lower solar radiation values because of persistent cloud cover and high atmospheric moisture associated with its coastal rainforest climate. Clouds significantly reduce solar radiation reaching the surface through reflection and absorption processes. Consequently, the amount of radiation available for photosynthesis and surface heating is reduced in this region (Wild, 2012). Ibadan shows moderate solar radiation levels, reflecting its intermediate climatic characteristics. Seasonal changes in cloud cover associated with the West African monsoon system contribute to fluctuations in solar radiation throughout the year. Overall, the observed trends across Kano, Ibadan, and Onne highlight clear spatial variations in temperature and solar radiation driven by regional climatic conditions. Northern Nigeria, represented by Kano, experiences higher solar radiation and maximum temperatures due to clearer skies and lower humidity. In contrast, the southern coastal region represented by Onne experiences lower solar radiation and moderated temperatures because of persistent cloud cover and high atmospheric moisture. Ibadan occupies an intermediate position with moderate climatic conditions and seasonal variability. These findings emphasize the importance of satellite-based datasets such as the NASA POWER system for understanding regional climate variability and supporting agricultural and environmental research. Since temperature and solar radiation strongly influence plant growth, evapotranspiration, and ecosystem productivity, understanding their spatial and temporal patterns is essential for climate adaptation and sustainable resource management.

### 3.4 Relationships between the NASA POWER variables and solar radiation

The correlation matrix (Table 1) presents the Pearson correlation coefficients and corresponding *p*-values describing the relationships between all-sky surface total Photosynthetically Active Radiation (PAR) and selected climatic variables, including cloud amount (CLOUD_AMT), relative humidity (RHM), maximum temperature (Tmax), minimum temperature (Tmin), and solar radiation (SR). All correlations were statistically significant at *p* < 0.05, indicating strong evidence of relationships among the variables. The results indicate a strong positive correlation between PAR and maximum temperature (r = 0.741). This suggests that periods with higher maximum temperatures are generally associated with increased PAR availability. Higher temperatures typically occur during clear sky conditions, allowing more incoming solar radiation to reach the Earth’s surface, thereby increasing PAR levels (Jones, 2014). Similarly, solar radiation (SR) showed a strong positive relationship with PAR (r = 0.739). This is expected because PAR represents the portion of solar radiation within the 400–700 nm wavelength range used in photosynthesis, meaning both variables are closely linked in atmospheric radiation processes (Monteith and Unsworth, 2013). Increased solar radiation therefore directly enhances PAR availability.

**Table 1:** Relationship between the NASA POWER parameters and measured solar radiation.

Parameters	All sky PAR	Cloud amount	RHM	Tmax	Tmin
Cloud amount	−0.571****				
RHM	−0.573****	0.756****			
Tmax	0.741****	−0.493****	−0.76****		
Tmin	−0.353****	0.806****	0.81****	−0.353****	
SR	0.739****	−0.583****	−0.705****	0.713****	−0.519****

***means significance at 0.0001.

Conversely, cloud amount (CLOUD_AMT) displayed a strong negative correlation with PAR (r = −0.571). Clouds act as a barrier that reduces the amount of solar radiation reaching the surface through reflection and absorption processes. Consequently, increased cloud cover leads to reduced PAR levels. Similar relationships have been documented in previous climate and radiation studies (Wild, 2012). Relative humidity (RHM) also exhibited a negative correlation with PAR (r = −0.573). Higher humidity levels are often associated with cloud formation and atmospheric moisture, both of which can scatter or absorb incoming radiation. This reduces the amount of usable PAR reaching plant canopies. The correlation between minimum temperature (Tmin) and PAR was moderately negative (r = −0.353). This may reflect nighttime cooling conditions or cloud-induced thermal regulation, where higher Tmin values may correspond with cloudy conditions that simultaneously reduce incoming radiation during the day. Strong relationships were also observed among the meteorological variables themselves. For example, relative humidity and minimum temperature showed a strong positive correlation (r = 0.810), indicating that humid atmospheric conditions are often associated with higher nighttime temperatures due to reduced radiative cooling. Additionally, cloud amount was strongly positively correlated with minimum temperature (r = 0.806), further supporting the influence of cloud cover on thermal regulation. Overall, the results demonstrate that solar radiation variability is strongly related to PAR, temperature, humidity, and cloud cover.

### 3.5 Performance evaluation of the developed model for solar radiation prediction during training and validation

Tables 2, 3 present the training and validation error metrics for solar radiation prediction models, including several variants of Gaussian Process Regression (GPR) and Support Vector Machine (SVM) algorithms. The performance of the models was evaluated using standard statistical indicators such as Root Mean Square Error (RMSE), Mean Squared Error (MSE), Mean Absolute Error (MAE), Normalized Root Mean Square Error (NRMSE), and the coefficient of determination (*R*^2^). These metrics are widely used to evaluate predictive model performance because they provide insight into the accuracy, precision, and reliability of machine learning models in environmental and solar radiation studies (Willmott and Matsuura, 2005; Chai and Draxler, 2014).

**Table 2:** Training error metrics result for the solar radiation prediction.

Model algorithms	Observed	RMSE	*R* ^2^	MSE	MAE	NRMSE
Models	16.51					
Rational quadratic GPR		2.03	0.74	4.13	1.61	0.123
Squared exponential GPR		2.06	0.73	4.24	1.63	0.125
Matern 5/2 GPR		2.04	0.74	4.15	1.1	0.124
Exponential GPR		2.01	0.74	4.06	1.59	0.122
Medium SVM		2.09	0.73	4.36	1.66	0.127
Fine SVM		2.23	0.69	4.96	1.72	0.135

**Table 3:** Validation error metrics result for the solar radiation prediction.

Model algorithms	Observed	RMSE	*R* ^2^	MSE	MAE	NRMSE
Models	15.37					
Rational quadratic GPR		2.51	0.68	6.31	1.71	0.163
Squared exponential GPR		2.52	0.68	6.37	1.71	0.164
Matern 5/2 GPR		2.53	0.68	6.4	1.71	0.165
Exponential GPR		2.51	0.68	6.31	1.7	0.163
Medium SVM		2.57	0.66	6.59	1.75	0.167
Fine SVM		3.05	0.53	9.29	2.39	0.198

Table 2 summarizes the training error metrics for solar radiation prediction. The observed solar radiation value during training was 16.51, and the results show relatively close error metric values across the different models. Among the tested models, the Exponential Gaussian Process Regression (GPR) model produced the lowest RMSE value of 2.01, indicating better predictive accuracy during training compared with the other models. Lower RMSE values generally imply that the predicted values are closer to the observed data, thereby reflecting improved model performance (Willmott and Matsuura, 2005). Similarly, the Exponential GPR model recorded the lowest MSE value (4.06) and a relatively low MAE value (1.59), suggesting that this model effectively minimized both squared and absolute prediction errors. The NRMSE value of 0.122 further indicates that the model achieved good normalization relative to the range of observed values, highlighting its stability and predictive precision. Other Gaussian Process Regression models, including Rational Quadratic GPR, Squared Exponential GPR, and Matern 5/2 GPR, also demonstrated strong predictive capability with RMSE values ranging from 2.03 to 2.06 and *R*^2^ values around 0.73–0.74. These results suggest that the GPR models explain approximately 73–74% of the variance in solar radiation, indicating strong predictive relationships between the input variables and the predicted radiation values. GPR models are widely recognized for their ability to capture nonlinear relationships and uncertainty in environmental datasets (Rasmussen and Williams, 2006). In comparison, the Support Vector Machine models, particularly the Fine SVM, exhibited relatively weaker performance during training. The Fine SVM recorded the highest RMSE (2.23), highest MSE (4.96), and highest NRMSE (0.135), indicating reduced predictive accuracy relative to the GPR models. Although the Medium SVM performed slightly better than the Fine SVM, its RMSE (2.09) and MAE (1.66) values were still higher than those of most GPR models. These findings suggest that the GPR models are more suitable for capturing the complex relationships involved in solar radiation prediction.

Table 3 presents the validation error metrics, which assess the ability of the models to generalize to unseen data. The observed solar radiation value for validation was 15.37. As expected, the validation errors are slightly higher than the training errors, which is typical in predictive modeling due to the model being tested on independent data. Among the models evaluated, the Rational Quadratic GPR and Exponential GPR models performed best during validation, both producing the lowest RMSE value of 2.51 and the lowest NRMSE value of 0.163. These results indicate that the models maintained good predictive capability even when applied to new datasets. The *R*^2^ value of 0.68 suggests that approximately 68% of the variance in solar radiation values is explained by the models, demonstrating reasonable predictive strength. The Squared Exponential GPR and Matern 5/2 GPR models also showed similar performance with RMSE values around 2.52–2.53 and MAE values of approximately 1.71, indicating consistent model accuracy across the GPR family. Such consistency further highlights the robustness of Gaussian Process Regression techniques for modeling environmental variables.

In contrast, the Fine SVM model performed considerably worse during validation, with the highest RMSE value of 3.05, the highest MSE value of 9.29, and the highest NRMSE value of 0.198. The *R*^2^ value of 0.53 indicates that the model explained only about 53% of the variance in the validation dataset, reflecting lower predictive accuracy and weaker generalization capability. This suggests that the Fine SVM model may have experienced some degree of overfitting during training or may not adequately capture the nonlinear relationships present in the solar radiation dataset. Overall, the results presented in Tables 2, 3 demonstrate that Gaussian Process Regression models outperform Support Vector Machine models in predicting solar radiation. The relatively lower RMSE, MSE, MAE, and NRMSE values obtained by the GPR models indicate higher accuracy and precision. Additionally, the stable *R*^2^ values observed during both training and validation suggest good model reliability and generalization capability. Accurate prediction of solar radiation is essential for renewable energy planning, agricultural productivity assessment, and climate modeling. Machine learning techniques such as GPR have increasingly been applied in solar radiation modeling because they effectively capture nonlinear relationships between meteorological variables and radiation levels (Rasmussen and Williams, 2006). The Normalized Root Mean Square Error (NRMSE) recorded in this study is lower than a value of 0.387 reported by Alkahtani et al. (2023) who applied long short-term memory (CNN-LSTM) to predict solar radiation. The R-square value obtained in this study compare favourably well with a value of 0.70 reported by Chaibi et al. (2022) using the Bayesian optimization technique and a value of 0.68 reported by Ke et al. (2017). However, some researchers (Chaibi et al., 2022; Faisal et al., 2022) reported higher values of R-square of 90% using Random Forest and Nonlinear autoregressive.

network (NAR). The higher R-square value reported by these researchers can be attributed to the fact that ground based meteorological data were used as input. Alternatively, it could be attributed to the fact that the prediction was carried out in a region with homogenous or similar climatic condition, while extremely contrasting climatic zones were considered in our study. The robustness of using the GPR and SVR algorithms applied in predicting the solar radiation is confirmed by lower mean square error (MSE) values obtained in our study compare to a value of 9.58 reported by Ke et al. (2017) who used Multi-step CNN stacked LSTM for the prediction of the solar radiation. Overall, during training and validation all predictions were ranked good because the NRMSE ranged between 10 and 20%.

### 3.6 Performance evaluation of the developed model for solar radiation prediction in all the three locations

Tables 4–6 present the performance evaluation metrics for solar radiation prediction models applied to three Nigerian locations: Kano, Ibadan, and Onne. The predictive performance of the models was assessed using standard statistical error metrics including Root Mean Square Error (RMSE), Mean Squared Error (MSE), Mean Absolute Error (MAE), and Normalized Root Mean Square Error (NRMSE). Table 4 presents the performance metrics for solar radiation prediction in Kano, where the observed solar radiation value was 20.68. Kano is located in the semi-arid northern region of Nigeria, characterized by relatively high solar radiation due to lower cloud cover and reduced atmospheric humidity. The results show that the Fine SVM model produced the lowest error values, including an RMSE of 2.16, MSE of 4.67, MAE of 1.43, and NRMSE of 0.104. These values indicate that the Fine SVM model achieved the highest prediction accuracy and precision for Kano among the evaluated models. The Exponential GPR model also demonstrated strong predictive capability, with an RMSE of 2.26 and NRMSE of 0.109, indicating relatively small prediction errors. The Rational Quadratic GPR and Matern 5/2 GPR models produced slightly higher error values, suggesting moderate predictive accuracy. The ability of the Fine SVM model to perform well in Kano may be attributed to the relatively stable atmospheric conditions and strong solar radiation patterns typical of semi-arid environments, which simplify the modeling process. Overall, model performance in Kano revealed that the predictions were ranked good because the NRMSE ranged between 10 and 20%.

**Table 4:** Performance evaluation metrics for solar radiation prediction for Kano location.

Model algorithms	Observed	Model	RMSE	MSE	MAE	NRMSE
Models						
Rational quadratic GPR	20.68	20.53	2.37	5.62	1.67	0.114
Squared exponential GPR	20.68	20.48	2.58	6.64	1.89	0.124
Matern 5/2 GPR	20.68	20.52	2.53	6.42	1.83	0.122
Exponential GPR	20.68	20.61	2.26	5.12	1.51	0.109
Medium SVM	20.68	20.32	2.7	7.3	1.99	0.131
Fine SVM	20.68	20.11	2.16	4.67	1.43	0.104

**Table 5:** Performance evaluation metrics for solar radiation prediction for Ibadan location.

Model algorithms	Observed	Model	RMSE	MSE	MAE	NRMSE
Models						
Rational quadratic GPR	15.16	15.26	1.69	2.86	1.35	0.111
Squared exponential GPR	15.16	15.21	1.83	3.36	1.49	0.121
Matern 5/2 GPR	15.16	15.22	1.8	3.23	1.46	0.119
Exponential GPR	15.16	15.23	1.46	2.14	1.17	0.096
Medium SVM	15.16	15.19	1.91	3.64	1.53	0.126
Fine SVM	15.16	15.64	1.93	3.74	1.33	0.128

**Table 6:** Performance evaluation metrics for solar radiation prediction for Onne location.

Model algorithms	Observed	Model	RMSE	MSE	MAE	NRMSE
Models						
Rational quadratic GPR	12.66	12.86	1.51	2.28	1.1	0.119
Squared exponential GPR	12.66	12.91	1.62	2.63	1.18	0.128
Matern 5/2 GPR	12.66	12.87	1.59	2.54	1.16	0.126
Exponential GPR	12.66	12.81	1.36	1.84	0.97	0.107
Medium SVM	12.66	13.01	1.64	2.68	1.18	0.129
Fine SVM	12.66	13.34	1.75	3.07	1.17	0.138

Table 5 presents the performance evaluation metrics for solar radiation prediction in Ibadan, where the observed solar radiation value was 15.16. Ibadan lies within the forest–savanna transition zone in southwestern Nigeria and experiences moderate solar radiation levels influenced by seasonal variations in cloud cover associated with the West African monsoon. The results indicate that the Exponential GPR model produced the best predictive performance for Ibadan, with the lowest RMSE value of 1.46, MSE of 2.14, MAE of 1.17, and NRMSE of 0.096. These low error values demonstrate high model accuracy and reliability. The ability of the Exponential GPR model to capture nonlinear relationships between meteorological variables likely contributed to its superior performance. Other GPR models, including Rational Quadratic GPR and Matern 5/2 GPR, also performed reasonably well, with RMSE values ranging between 1.69 and 1.80. In contrast, the SVM models showed relatively higher error values, indicating slightly lower predictive accuracy. The Medium SVM model recorded an RMSE of 1.91, while the Fine SVM model produced an RMSE of 1.93. These results suggest that GPR models are better suited for modeling solar radiation patterns in regions with moderate climatic variability such as Ibadan. Overall, model performance in Ibadan revealed that the predictions were ranked good because the NRMSE ranged between 10 and 20%, expect for Exponential GPR, which produced an excellent prediction with NRMSE < 10%.

Table 6 presents the performance metrics for solar radiation prediction in Onne, where the observed solar radiation value was 12.66. Onne is located in the humid coastal region of southern Nigeria within the Niger Delta. This region experiences high cloud cover, high relative humidity, and frequent rainfall, which introduce significant variability in solar radiation patterns. Among the evaluated models, the Exponential GPR model again demonstrated the best performance, with the lowest RMSE value of 1.36, MSE of 1.84, MAE of 0.97, and NRMSE of 0.107. These values indicate a high level of prediction accuracy and model precision. The strong performance of the Exponential GPR model suggests that it effectively captured the complex nonlinear relationships between atmospheric variables and solar radiation in humid environments. Other GPR models also produced relatively low error values, indicating reliable predictions. In contrast, the Fine SVM model recorded the highest RMSE value (1.75) and highest NRMSE (0.138), indicating lower predictive accuracy compared with the GPR models. The relatively weaker performance of SVM models in Onne may be due to the complex atmospheric interactions present in humid tropical climates, including high cloud variability and moisture content. Across the three locations, the results reveal clear differences in model performance depending on regional climatic conditions. The Fine SVM model performed best in Kano, whereas the Exponential GPR model consistently produced the most accurate predictions for Ibadan and Onne. The relatively lower RMSE, MSE, MAE, and NRMSE values obtained by the Exponential GPR model indicate improved prediction accuracy and reliability. Overall, the findings demonstrate that Gaussian Process Regression models are generally more effective for solar radiation prediction, particularly in regions with complex atmospheric conditions. Their ability to capture nonlinear relationships and uncertainty makes them suitable for environmental modeling applications (Rasmussen and Williams, 2006). Overall, model performance in Onne revealed that the predictions were ranked good because the NRMSE ranged between 10 and 20%.

One major factor is the high atmospheric variability in humid regions. In tropical coastal climates like Onne, solar radiation reaching the Earth’s surface is strongly influenced by rapidly changing meteorological factors such as cloud dynamics, atmospheric moisture, and convective processes. These factors create nonlinear interactions between input variables and solar radiation levels. Traditional deterministic models often struggle to capture these complex relationships. However, GPR is a probabilistic machine learning method that can effectively model nonlinear dependencies and adapt to complex environmental data structures (Rasmussen and Williams, 2006). Another reason for the strong performance of GPR in wetter regions is its ability to quantify uncertainty in predictions. Unlike many machine learning methods that provide only point predictions, GPR produces both a predicted mean and a confidence interval for each prediction. This characteristic makes GPR particularly suitable for modeling environmental processes where uncertainty is inherent due to fluctuating atmospheric conditions (Murphy, 2012). In humid regions where cloud formation can rapidly alter solar radiation levels, the probabilistic nature of GPR allows it to better account for such uncertainties. Additionally, wetter regions typically exhibit higher levels of noise and variability in meteorological data. Cloud cover and humidity introduce more irregular fluctuations in solar radiation measurements in the southern region than in the Northern region (Table 7). This can be confirmed by the highest coefficient of variation observed at Onne location.

**Table 7:** Descriptive statistics of the climate variables.

Location	Parameters	ALLSKY_SFC_PAR_TOT	CLOUD_AMT	RHM	Tmax	Tmin	SR
Kano	Average	10.07	47.25	45.75	37.69	16.00	20.68
	STD	0.85	15.56	20.77	2.88	4.80	2.61
	CV	0.085	0.33	0.45	0.077	0.30	0.13
Ibadan	Average	8.92	62.18	76.95	32.92	19.72	15.16
	STD	0.93	18.22	11.57	2.67	2.89	2.46
	CV	0.10	0.29	0.15	0.081	0.15	0.16
Onne	Average	7.71	74.21	87.85	30.34	22.17	12.66
	STD	1.20	14.99	4.50	1.25	1.91	2.12
	CV	0.16	0.20	0.051	0.041	0.086	0.17

STD is standard deviation, CV is coefficient of variation, RHM is relative humidity, Tmax is maximum air temperature, Tmin is minimum air temperature, and SR is the solar radiation.

Gaussian Process models are known for their ability to handle noisy datasets through the use of covariance (kernel) functions that capture spatial and temporal relationships within the data (Rasmussen and Williams, 2006). These kernel functions allow GPR to model subtle variations in atmospheric conditions that strongly influence solar radiation in humid climates. The flexibility of GPR kernel functions is another important factor. Kernels such as the Exponential, Rational Quadratic, and Matern kernels can model different degrees of smoothness and variability in environmental data. In wetter regions where solar radiation changes most irregularly due to cloud formation and atmospheric moisture (as evidenced in Table 7), these kernels enable GPR models to capture complex patterns more effectively than algorithms that rely on fixed decision boundaries, such as Support Vector Machines (SVM).

Furthermore, studies on solar radiation modeling have demonstrated that probabilistic and nonlinear machine learning models tend to perform better in regions with high climatic variability, with respect to the solar radiation. Humid tropical climates often show strong interactions between solar radiation, relative humidity, cloud amount, and temperature. GPR models can incorporate these interactions within their covariance structure, allowing them to produce more accurate predictions in such environments (Mellit and Kalogirou, 2008). The climatic characteristics of southern Nigeria help explain these findings. Regions such as Onne experience high relative humidity, persistent cloud cover, and strong maritime influence from the Atlantic Ocean. These conditions produce highly variable solar radiation patterns. The ability of GPR models to capture nonlinear relationships and incorporate uncertainty therefore makes them particularly effective for solar radiation prediction in these wetter environments.

In contrast, drier regions such as Kano generally exhibit more stable atmospheric conditions with lower solar radiation variability. In such environments, simpler machine learning models like Support Vector Machines can perform well because the relationship between meteorological variables and solar radiation is more predictable. Kano exhibits relatively lower atmospheric variability, clearer skies, and reduced cloud-cover fluctuations, resulting in smoother relationships between predictor variables and solar radiation that are effectively captured by SVM (Álvarez-Alvarado et al., 2021). Conversely, the humid regions experience greater cloud variability, aerosol loading, atmospheric moisture fluctuations, and increased stochastic uncertainty. Gaussian Process Regression explicitly models predictive uncertainty through covariance functions, making it better suited for highly variable environmental conditions (Rasmussen and Williams, 2006). Overall, the results of this study suggest that model performance is strongly influenced by regional climatic characteristics. Gaussian Process Regression models are better suited for humid environments with complex atmospheric dynamics, while models like SVM may perform adequately in more stable dry climates. Understanding these differences is important for selecting appropriate predictive models in solar radiation forecasting and renewable energy resource assessment. Accurate solar radiation prediction is essential for renewable energy planning, agricultural productivity assessment, and climate research, particularly in regions such as Nigeria where solar energy potential is high.

### 3.7 Uncertainty quantification for solar radiation prediction in the study area

Table 8 presents the uncertainty quantification of the machine learning models used for solar radiation prediction in Kano, Ibadan, and Onne using confidence interval (margin of error) values at a 95% confidence level (*α* = 0.05). Uncertainty quantification is an essential component of predictive modeling because it evaluates the reliability of model outputs by estimating the range within which the true value is expected to lie with a specified probability (Almawgani et al., 2025; Abdulkarem et al., 2026). Unlike conventional performance indices such as RMSE, MAE, and MSE, which measure prediction error, confidence intervals quantify the precision and uncertainty associated with model predictions (Rasmussen and Williams, 2006; Murphy, 2012). Models with smaller confidence intervals are generally considered more precise because their predictions exhibit less variability and greater consistency.

**Table 8:** Confidence interval value for the output predicted by the different modelling algorithm.

Modelling algorithm	Confidence value (Margin of error)		
	Kano	Ibadan	Onne
Rational GPR	0.2447	0.2248	0.1905
Squared GPR	0.2452	0.2307	0.1969
Matern GPR	0.2466	0.2296	0.1954
Exponential GPR	0.2485	0.2238	0.1895
Medium SVR	0.2388	0.2253	0.1952
Fine SVR	0.2244	0.2260	0.2037
Sample size	288	288	288
Level of significance	0.05	0.05	0.05

For Kano, the confidence interval values ranged from 0.2244 to 0.2485, indicating relatively low prediction uncertainty across all the evaluated models. The Fine Support Vector Regression (Fine SVR) produced the smallest margin of error (0.2244), suggesting that it generated the most precise and reliable predictions in the semi-arid environment. This finding is consistent with the earlier error metric analysis, where Fine SVR recorded the lowest RMSE, MAE, and NRMSE values for Kano. The relatively stable atmospheric conditions in northern Nigeria, characterized by lower humidity, fewer clouds, and more consistent solar radiation patterns, likely reduced the variability in the prediction process, allowing Fine SVR to achieve the highest prediction precision. Conversely, the Exponential Gaussian Process Regression (Exponential GPR) recorded the highest confidence interval (0.2485), indicating slightly greater uncertainty. However, the difference between the confidence intervals of the models is relatively small (approximately 0.024), demonstrating that all models produced reliable predictions under the climatic conditions of Kano.

For Ibadan, the uncertainty values ranged between 0.2238 and 0.2307, which are lower than those obtained for Kano. The Exponential GPR achieved the lowest confidence interval (0.2238), followed closely by Rational Quadratic GPR (0.2248) and Medium SVR (0.2253). These narrow margins of error indicate high prediction precision and suggest that the Gaussian Process Regression models effectively captured the moderate climatic variability associated with the forest–savanna transition zone. Ibadan experiences seasonal fluctuations in cloud cover and atmospheric moisture resulting from the interaction between tropical maritime and continental air masses. The probabilistic framework of GPR enables it to represent these nonlinear atmospheric processes while simultaneously estimating predictive uncertainty, thereby improving the reliability of solar radiation predictions (Rasmussen and Williams, 2006). The Fine SVR model recorded a confidence interval of 0.2260, only marginally higher than the best-performing models, indicating that SVM also maintained acceptable prediction precision in the transitional climatic environment.

The lowest uncertainty values among all study locations were observed in Onne, where confidence intervals ranged from 0.1895 to 0.2037. The Exponential GPR again produced the smallest margin of error (0.1895), followed by Rational Quadratic GPR (0.1905). These results demonstrate that GPR models generated the most precise predictions in the humid coastal environment. Onne is characterized by persistent cloud cover, high relative humidity, and frequent rainfall, which create highly nonlinear relationships between meteorological variables and solar radiation. Gaussian Process Regression is particularly suited to such environments because it models both the prediction function and the associated uncertainty through covariance (kernel) functions, enabling it to account for atmospheric variability more effectively than deterministic approaches (Murphy, 2012; Rasmussen and Williams, 2006). In contrast, Fine SVR recorded the highest confidence interval (0.2037), indicating greater uncertainty in comparison with the GPR models. This result agrees with the error metric analysis presented earlier, where Fine SVR also exhibited relatively poorer prediction performance in the humid environment, suggesting that deterministic kernel-based regression may be less capable of capturing the complex atmospheric dynamics associated with high humidity and cloud variability.

A comparison across the three locations reveals clear climatic influences on predictive uncertainty. Although Fine SVR produced the narrowest confidence interval in Kano, the Exponential GPR consistently yielded the smallest confidence intervals in both Ibadan and Onne. These findings indicate that Support ssVector Regression performs better under relatively stable atmospheric conditions, whereas Gaussian Process Regression provides more reliable predictions in environments characterized by higher climatic variability and uncertainty. This observation is consistent with the theoretical properties of GPR, which explicitly models predictive variance and therefore adapts more effectively to complex nonlinear environmental processes (Rasmussen and Williams, 2006). The use of 288 observations for each study location further enhances the credibility of the uncertainty estimates. Adequate sample size improves the stability of statistical estimates and reduces sampling variability, resulting in more dependable confidence intervals (Montgomery et al., 2021). Moreover, the adoption of a 5% level of significance (95% confidence interval) conforms to international statistical practice and indicates that there is only a 5% probability that the true solar radiation value falls outside the reported confidence limits. This provides a high degree of confidence in the predictive capability of the developed machine learning models.

Overall, the uncertainty quantification confirms that all the evaluated machine learning algorithms produced relatively narrow confidence intervals, indicating good predictive reliability. Nevertheless, the results demonstrate that the most appropriate model depends on the prevailing climatic conditions. Fine SVR exhibited the greatest prediction precision in the semi-arid climate of Kano, while Exponential GPR consistently achieved the lowest uncertainty in the wetter environments of Ibadan and Onne. These findings complement the error metric analysis and further demonstrate that incorporating uncertainty quantification alongside conventional accuracy measures provides a more comprehensive assessment of machine learning model performance for solar radiation prediction. Such an integrated evaluation is particularly important for renewable energy planning, evapotranspiration estimation, and agricultural decision support, where confidence in model predictions is as important as prediction accuracy.

## 4 Conclusion

This study evaluated the performance of different machine learning models for solar radiation prediction across three distinct climatic regions in Nigeria: Kano, Ibadan, and Onne using meteorological data obtained from the NASA POWER. The predictive capability of several Gaussian Process Regression (GPR) models and Support Vector Machine (SVM) models was assessed using standard statistical error metrics including RMSE, MSE, MAE, NRMSE, and the coefficient of determination (*R*^2^). The results demonstrated that machine learning techniques are effective tools for modeling solar radiation in different climatic environments. Among the evaluated models, Gaussian Process Regression (GPR) models generally produced the most reliable and accurate predictions across the study locations. In particular, the Exponential GPR model consistently achieved the lowest error metrics for Ibadan and Onne, indicating superior predictive performance in humid and coastal environments characterized by higher solar radiation variability. The probabilistic nature and nonlinear modeling capability of GPR enable it to effectively capture the complex interactions between meteorological variables and solar radiation in such regions.

Conversely, the Fine Support Vector Machine (SVM) model produced the best predictive performance in Kano, which represents a semi-arid climate with relatively stable atmospheric conditions, in respect of the solar radiation trend. The simpler and more predictable solar radiation patterns in this region likely contributed to the improved performance of the SVM model. Overall, the findings of this study highlight that model performance for solar radiation prediction is strongly influenced by regional climatic conditions. While SVM models may perform well in relatively stable dry environments, GPR models appear to be more suitable for regions with higher atmospheric variability such as humid tropical climates. The study therefore demonstrates the potential of machine learning approaches for improving solar radiation prediction and renewable energy resource assessment in Nigeria. The results can support solar energy planning, climate analysis, and agricultural decision-making by providing more accurate estimates of solar radiation across different ecological zones. Future studies may further improve prediction accuracy by incorporating additional climatic variables and exploring advanced hybrid machine learning techniques. The present study utilized historical observations ending in 2007 because they constitute a complete and quality-controlled dataset available for the selected stations. Nevertheless, future investigations should incorporate recent datasets, such as NASA POWER or other contemporary satellite-derived observations, to evaluate model performance under present-day climatic conditions and potential climate-change influences.

## Data Availability

The raw data supporting the conclusions of this article will be made available by the authors, without undue reservation.

## Footnotes

1
https://power.larc.nasa.gov/
